# Thyroid hormone replacement is associated with improvement in renal dysfunction in patients with hypothyroidism: a retrospective observational study

**DOI:** 10.3389/fendo.2026.1840954

**Published:** 2026-08-24

**Authors:** Teng Zhao, Nan Zhang, Yong Tian, Zhengqiang Yang

**Affiliations:** Department of Endocrinology, Beijing Huairou Hospital, Beijing, China

**Keywords:** blood urea nitrogen, estimated glomerular filtration rate, hypothyroidism, lipid profile, renal dysfunction, thyroid

## Abstract

**Background:**

Hypothyroidism is a common endocrine disorder with multisystem involvement and is increasingly recognized as a potentially reversible contributor to renal dysfunction. However, renal impairment related to thyroid hormone deficiency is frequently overlooked or misclassified as chronic kidney disease in clinical practice.

**Methods:**

In this single-center retrospective observational study, 40 hospitalized adults with newly diagnosed hypothyroidism were included. Renal dysfunction was defined by elevated serum creatinine and reduced estimated glomerular filtration rate at admission. All patients received levothyroxine replacement therapy as part of routine care. Biochemical parameters were assessed at three timepoints: admission (T0), normalization of thyroid-stimulating hormone (T1), and normalization of serum creatinine (T2). Serum creatinine was the primary renal outcome, whereas estimated glomerular filtration rate was evaluated as a supportive calculated indicator. Secondary outcomes included thyroid hormones and other biochemical markers.

**Results:**

At baseline, mean serum creatinine was 114.88 ± 25.61 μmol/L and estimated glomerular filtration rate was 51.42 ± 16.32 mL/min/1.73 m². Following treatment, serum creatinine decreased significantly to 85.13 ± 21.01 μmol/L at T1 and 79.92 ± 5.64 μmol/L at T2 (both P < 0.01). Estimated glomerular filtration rate improved to 74.48 ± 32.56 and 75.04 ± 14.52 mL/min/1.73 m², respectively. Free thyroid hormone levels increased, while other biochemical parameters showed no consistent changes. Analyses at T1 and T2 were based on responder subgroups that achieved predefined biochemical targets.

**Conclusion:**

Thyroid hormone replacement was associated with significant short-term improvement in creatinine-based renal indices, suggesting that renal dysfunction in hypothyroidism may be at least partially functional and potentially reversible in some patients. These findings should be interpreted cautiously given the small sample size, retrospective design, responder-based subgroup analyses, absence of a comparison group, and potential residual confounding.

## Introduction

Hypothyroidism is a clinical syndrome caused by insufficient synthesis, secretion, or biological action of thyroid hormones, resulting in multisystem metabolic and hemodynamic disturbances (1). Because its clinical manifestations are often subtle and nonspecific, particularly in early or mild disease, hypothyroidism is frequently underdiagnosed or diagnosed late in the disease course. Although clinically overt renal dysfunction has been reported in approximately 2% of patients with hypothyroidism, this estimate is derived primarily from hospitalized patients with overt hypothyroidism and likely underestimates the true prevalence of early or subclinical renal involvement in broader clinical populations (2). If untreated, hypothyroidism may contribute to the progression of chronic kidney disease (CKD) through sustained alterations in renal perfusion, glomerular filtration, and metabolic homeostasis, emphasizing the importance of timely recognition and intervention (3). Thyroid hormones play a critical role in renal development, renal blood flow regulation, and tubular and glomerular function. Experimental studies have demonstrated that hyperthyroidism increases kidney-to-body weight ratios, whereas hypothyroidism is associated with reduced renal size and diminished renal perfusion (2–5). Clinical studies have likewise reported that levothyroxine replacement is associated with reductions in serum creatinine and improvement in estimated glomerular filtration rate (eGFR), although the magnitude of benefit varies across studies and the available evidence is derived predominantly from observational investigations with relatively few randomized controlled trials (4). More recently, a systematic review and meta-analysis by Wang et al. demonstrated that hypothyroidism is consistently associated with impaired renal function, while concluding that evidence supporting improvements in kidney function following levothyroxine replacement remains limited and heterogeneous, highlighting the need for well-designed prospective and randomized studies (6). These findings emphasize that, although thyroid hormone replacement may improve creatinine-based renal indices in some patients, the causal relationship and long-term renal benefits remain incompletely established. Autoimmune thyroiditis, particularly Hashimoto’s thyroiditis, represents the most common etiology of hypothyroidism and may indirectly affect renal function through immune-mediated and inflammatory mechanisms. Moreover, the coexistence of systemic autoimmune disorders, such as lupus nephritis, further underscores the complex interaction between thyroid dysfunction and renal pathology (7–9). In addition to its renal effects, hypothyroidism significantly influences lipid metabolism and cardiovascular function. Decreased low-density lipoprotein receptor activity leads to dyslipidemia, which can promote oxidative stress, endothelial dysfunction, and podocyte injury, thereby exacerbating renal damage (10–12). Elevated thyroid-stimulating hormone (TSH) levels have also been identified as an independent risk factor for atherosclerosis, potentially compounding renal microvascular impairment (13–15). Furthermore, reduced myocardial contractility and cardiac output in hypothyroid states impair effective renal perfusion, a pathophysiological mechanism that has been reported to improve following restoration of euthyroidism with thyroid hormone replacement (13, 14).

Collectively, these observations highlight a clinically relevant yet incompletely characterized issue: renal dysfunction associated with hypothyroidism appears potentially reversible, but the extent and temporal pattern of renal recovery remain uncertain. Furthermore, most published clinical studies are retrospective, involve relatively small cohorts, or lack appropriate comparison groups, leaving important uncertainties regarding the magnitude and causality of the observed renal changes following thyroid hormone replacement. In this single-center retrospective observational study, we evaluated changes in renal function among hospitalized patients with newly diagnosed hypothyroidism receiving levothyroxine as part of routine clinical care, with particular emphasis on serum creatinine (Scr) as the primary renal outcome and eGFR as a supportive calculated indicator of renal response. By examining biochemical parameters at baseline and during defined recovery stages, this study aims to describe the association between thyroid hormone replacement and short-term changes in renal indices and to inform clinical monitoring strategies in patients with endocrine disorders.

## Materials and methods

### Study design

**Figure 1:**
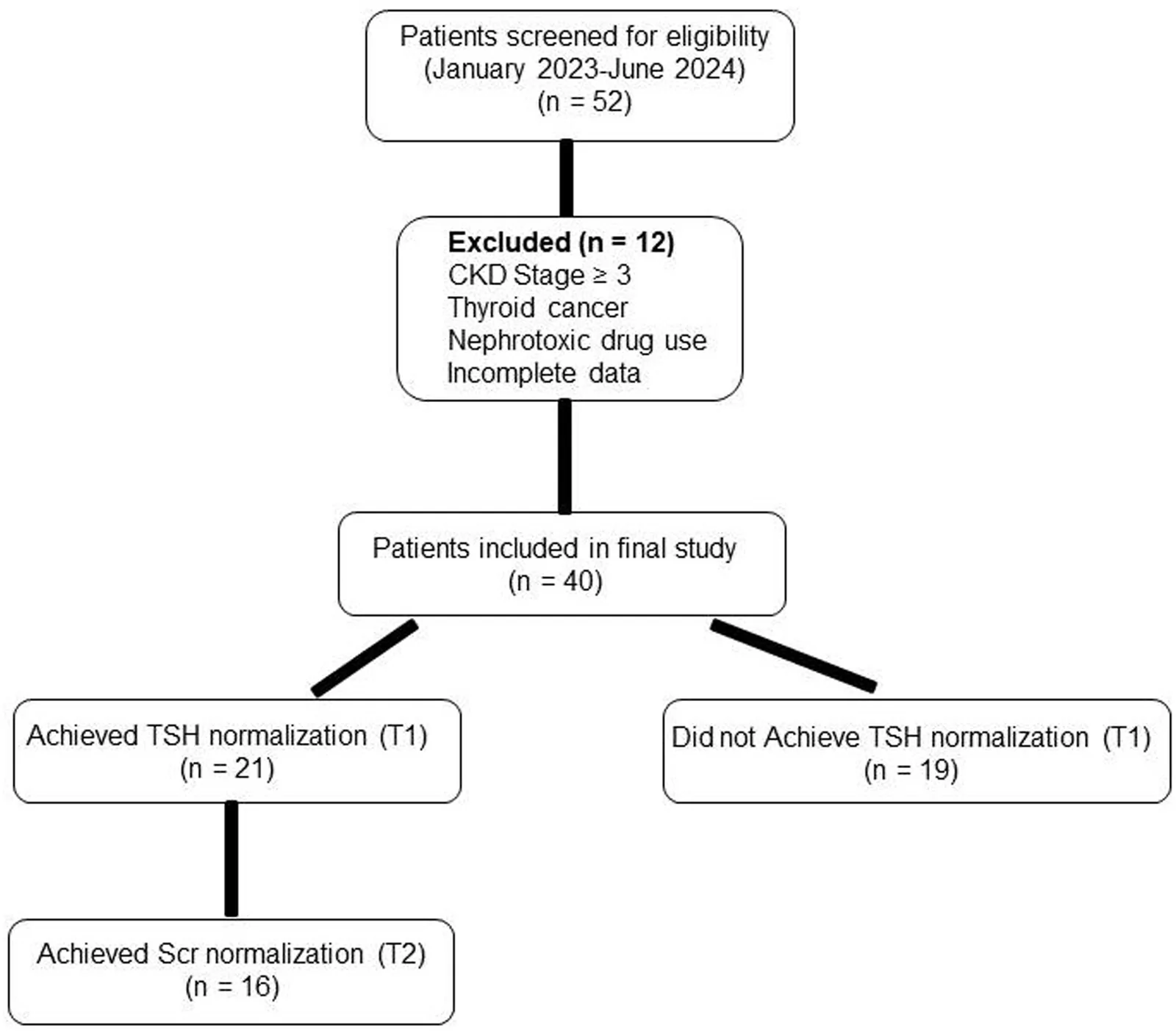
STROBE flow diagram of patient selection. Flowchart showing screening, exclusion, and final inclusion of patients with hypothyroidism, and subsequent subgroup classification according to achievement of TSH normalization (T1) and serum creatinine normalization (T2) during follow-up.

This retrospective observational study was conducted at Beijing Huairou Hospital between January 2023 and June 2024. During the study period, 52 consecutive adult patients with suspected hypothyroidism were initially identified through the hospital electronic medical record system using discharge diagnoses, endocrine consultation records, and laboratory records demonstrating elevated TSH levels. After screening, 12 patients were excluded based on predefined exclusion criteria, and a total of 40 patients with confirmed hypothyroidism were included in the final analysis. All included patients underwent clinical evaluation and levothyroxine treatment during hospitalization. The flow diagram of the study is presented in **Figure 1**. This study was designed and reported in accordance with the STROBE (Strengthening the Reporting of Observational Studies in Epidemiology) guidelines. This study followed a retrospective cohort framework with longitudinal assessment of biochemical parameters during treatment.

### Ethics statement

The study was conducted in accordance with the Declaration of Helsinki. The study protocol was reviewed and approved by the Research Ethics Committee of Beijing Huairou Hospital (Approval No. 2024-024-01). Due to the retrospective nature of the study, the use of anonymized clinical data, and the absence of any intervention beyond routine clinical care, the requirement for written informed consent was waived by the Ethics Committee.

### Patient identification and selection

Patients admitted with a confirmed diagnosis of hypothyroidism were eligible for inclusion. The diagnostic criterion for hypothyroidism was a thyroid-stimulating hormone (TSH) level exceeding 4.5 μIU/mL in conjunction with compatible clinical features. Overt hypothyroidism was defined as TSH >4.5 μIU/mL with free thyroxine (FT4) below the reference range (10–22 pmol/L), whereas subclinical hypothyroidism was defined as TSH >4.5 μIU/mL with normal FT4 levels. Patients with suspected central hypothyroidism were excluded based on clinical history and absence of inappropriately low or normal TSH in the setting of low FT4. The analyzed sample consisted of 40 patients at admission (T0), among whom 21 achieved biochemical normalization of TSH during follow-up (T1), and 16 subsequently achieved normalization of serum creatinine (Scr) levels (T2). Inclusion criteria comprised age ≥18 years, availability of complete thyroid and renal function data at relevant timepoints, and treatment with levothyroxine during hospitalization. Patients with chronic kidney disease stage ≥3, thyroid malignancy, or exposure to nephrotoxic medications unrelated to thyroid management were excluded. Chronic kidney disease stage ≥3 was defined as documented eGFR <60 mL/min/1.73 m² persisting for at least 3 months prior to admission. Mild chronic kidney disease (stage 1–2) was not an exclusion criterion and is reported as a comorbidity in **Table 1**. No patients were lost to follow-up during the study period. Timepoints T1 and T2 represent conditional subgroups defined by target achievement rather than repeated measures in the entire cohort, and therefore should be interpreted as responder-based analyses.

**Table 1:** Patient characteristics and biochemical indicators at admission, TSH normalization, and serum creatinine normalization.

Indicator	Reference range	Admission (n = 40)	TSH target (n = 21)	Scr target (n = 16)	P-value①	P-value②
Male/Female (cases)	–	10/30	7/14	6/10	–	–
Age (years)	–	72 ± 13	68 ± 16	70 ± 17	–	–
T3 (nmol/L)	1.0–2.0	0.80 ± 0.20	1.29 ± 0.43	1.20 ± 0.32	0.092	0.192
FT3 (pmol/L)	3.5–6.5	5.47 ± 3.37	5.71 ± 2.65	4.93 ± 3.10	0.006	0.017
T4 (nmol/L)	60–160	56.2 ± 8.2	74.4 ± 9.3	73.2 ± 5.5	0.217	0.366
FT4 (pmol/L)	10–22	7.10 ± 3.52	10.70 ± 1.18	9.22 ± 1.68	0.021	0.084
TSH (µIU/mL)	0.4–4.5	35.07 ± 24.21	3.34 ± 2.09	4.62 ± 3.58	0.005	0.005
Serum creatinine (µmol/L)	62–115	114.88 ± 25.61	85.13 ± 21.01	79.92 ± 5.64	0.001	<0.001
Blood urea nitrogen (mmol/L)	2.5–7.1	9.87 ± 5.14	6.77 ± 2.08	5.62 ± 1.12	0.493	0.199
Serum uric acid (µmol/L)	140–420	444.93 ± 175.38	357.03 ± 137.67	451.20 ± 141.93	0.237	0.626
eGFR (mL/min/1.73 m²)	>90	51.42 ± 16.32	74.48 ± 32.56	75.04 ± 14.52	0.040	0.001
ALT (U/L)	7–56	32.63 ± 53.00	22.00 ± 13.58	22.66 ± 12.10	0.385	0.438
AST (U/L)	10–40	34.46 ± 17.73	33.48 ± 17.25	36.04 ± 13.41	0.488	0.732
CK (U/L)	26–192	440.88 ± 742.80	105.15 ± 52.33	175.30 ± 149.30	0.245	0.386
CK-MB (U/L)	0–25	19.14 ± 15.30	14.15 ± 3.76	17.10 ± 6.39	0.163	0.314
Total cholesterol (mmol/L)	<5.2	5.72 ± 4.19	5.04 ± 1.04	4.38 ± 1.06	0.336	0.522
LDL-C (mmol/L)	<3.4	3.16 ± 2.51	2.80 ± 0.89	2.45 ± 0.97	0.323	0.824
HDL-C (mmol/L)	>1.0	1.22 ± 0.59	1.30 ± 0.22	1.18 ± 0.26	0.306	0.205
Triglycerides (mmol/L)	<1.7	1.63 ± 0.96	1.08 ± 0.45	1.24 ± 1.11	0.136	0.759
Hypothyroidism cause	–	Hashimoto’s thyroiditis (10), Post-surgical (4), Other/unspecified (26)	–	–	–	–
Antibody status	–	TPOAb positive (28), TgAb positive (26)	–	–	–	–
Ultrasound findings	–	Hypoechogenicity (24), Thyroid atrophy (8)	–	–	–	–
Comorbidities	–	Hypertension (25), Diabetes mellitus (15), CKD stage 1–2 (5)	–	–	–	–
Proteinuria	–	4 positive at baseline (dipstick); resolved during follow-up	–	–	–	–
HbA1c (%)	<5.7	7.1 ± 1.2	6.4 ± 0.8	6.8 ± 0.9	0.044	0.041
Blood pressure (mmHg)	120/80	142 ± 10/86 ± 8	136 ± 8/82 ± 7	132 ± 9/80 ± 6	0.037	0.045

Continuous variables are presented as mean ± standard deviation (SD) and were compared using paired t-tests or Wilcoxon signed-rank tests according to data distribution. Categorical clinical characteristics represent baseline values for the full cohort (n = 40). P-value①: Admission (T0) vs. TSH Target (T1); P-value②: Admission (T0) vs. Serum Creatinine Target (T2). CKD refers to stage 1–2 only; patients with CKD stage ≥3 were excluded from the study.

### Data collection and follow-up

Clinical and laboratory data were extracted from electronic medical records and hospital laboratory databases. Thyroid function parameters, including total triiodothyronine (T3), free triiodothyronine (FT3), total thyroxine (T4), free thyroxine (FT4), and TSH, were measured using chemiluminescent immunoassays on the Abbott Architect i2000SR system. Renal function indicators, including serum creatinine, blood urea nitrogen (BUN), and uric acid, were assessed using enzymatic methods on the Beckman Coulter AU5800 analyzer. Estimated glomerular filtration rate (eGFR) was calculated using the Modification of Diet in Renal Disease (MDRD) equation, which incorporates age and serum creatinine. Serum creatinine was prespecified as the primary renal outcome, and eGFR was treated as a calculated supportive indicator. Because eGFR is derived from serum creatinine, changes in eGFR were interpreted as supportive rather than independent outcomes.

Liver enzymes (alanine aminotransferase and aspartate aminotransferase), muscle enzymes (creatine kinase and creatine kinase-MB), and lipid parameters (triglycerides, total cholesterol, low-density lipoprotein cholesterol, and high-density lipoprotein cholesterol) were measured using standard automated biochemical assays in the hospital laboratory. Levothyroxine therapy was initiated as part of routine clinical care at individualized starting doses based on patient age, clinical status, cardiovascular risk, baseline thyroid function, and treating endocrinologist judgment. Elderly patients or those with cardiovascular comorbidities generally received lower initial doses (typically 25–50 μg/day), whereas younger patients without significant comorbidities could receive higher starting doses in accordance with routine clinical practice. Dose adjustments were performed every 1–2 weeks according to serial TSH measurements, clinical response, and standard endocrine management; weight-based dosing was not routinely applied because treatment was individualized in this retrospective clinical cohort. Patients were evaluated at three predefined timepoints: at admission before initiation of thyroid hormone therapy (T0), at the time of biochemical normalization of TSH to the reference range of 0.4–4.5 μIU/mL (T1; median 21 days, range 14–35 days), and at the time of normalization of serum creatinine (<110 μmol/L in males and <90 μmol/L in females; T2; median 32 days, range 21–49 days). Timepoints T1 and T2 represent target-achievement subsets rather than the entire cohort.

### Clinical parameters and comorbidities

Additional clinical data included thyroid antibody status (thyroglobulin antibody, thyroid peroxidase antibody, and TSH receptor antibody), thyroid ultrasonography findings, and documented comorbidities such as hypertension, diabetes mellitus, and coronary heart disease. Cardiac-related parameters, including hemoglobin concentration, B-type natriuretic peptide levels, left ventricular ejection fraction, presence of pericardial effusion, and arterial sclerosis, were recorded when available. Common hypothyroidism-related symptoms, including fatigue, edema, and dyspnea, were also documented. Proteinuria was assessed semi-quantitatively using urine dipstick testing during routine inpatient evaluation.

### Statistical analysis

Continuous variables were assessed for normality using visual inspection of histograms and Q–Q plots and the Shapiro–Wilk test. Variables with approximately normal distributions are presented as mean ± standard deviation, whereas skewed variables are presented as median (interquartile range). For within-patient comparisons between admission (T0) and follow-up timepoints, paired t-tests were used for normally distributed variables, and the Wilcoxon signed-rank test was applied for non-normally distributed variables. Because serum thyroid-stimulating hormone (TSH) and creatine kinase (CK) demonstrated markedly skewed distributions, nonparametric methods were applied for analyses involving these variables. Effect sizes are reported as mean differences with 95% confidence intervals for primary outcomes. To account for potential confounding, an exploratory multivariable linear regression analysis was performed in a subset of patients with complete longitudinal data (**Supplementary Table S1**). The dependent variable was the change in serum creatinine (ΔScr from baseline to follow-up), and independent variables included age, baseline serum creatinine, hypertension, and diabetes mellitus. Hydration status, inpatient fluid administration, and concomitant medication adjustments were not consistently available from the retrospective medical records and therefore could not be included in the regression model, representing potential sources of residual confounding. All statistical analyses and graphical visualizations were performed using R software (R Foundation for Statistical Computing, Vienna, Austria). A two-sided p-value <0.05 was considered statistically significant. No formal adjustment for multiple comparisons was performed given the exploratory nature of secondary endpoints; therefore, findings should be interpreted cautiously. Given the limited sample size and incomplete data at later timepoints, multivariable analysis was considered exploratory and hypothesis-generating rather than confirmatory.

## Results

### Renal function indicators

#### Serum creatinine

**Figure 2:**
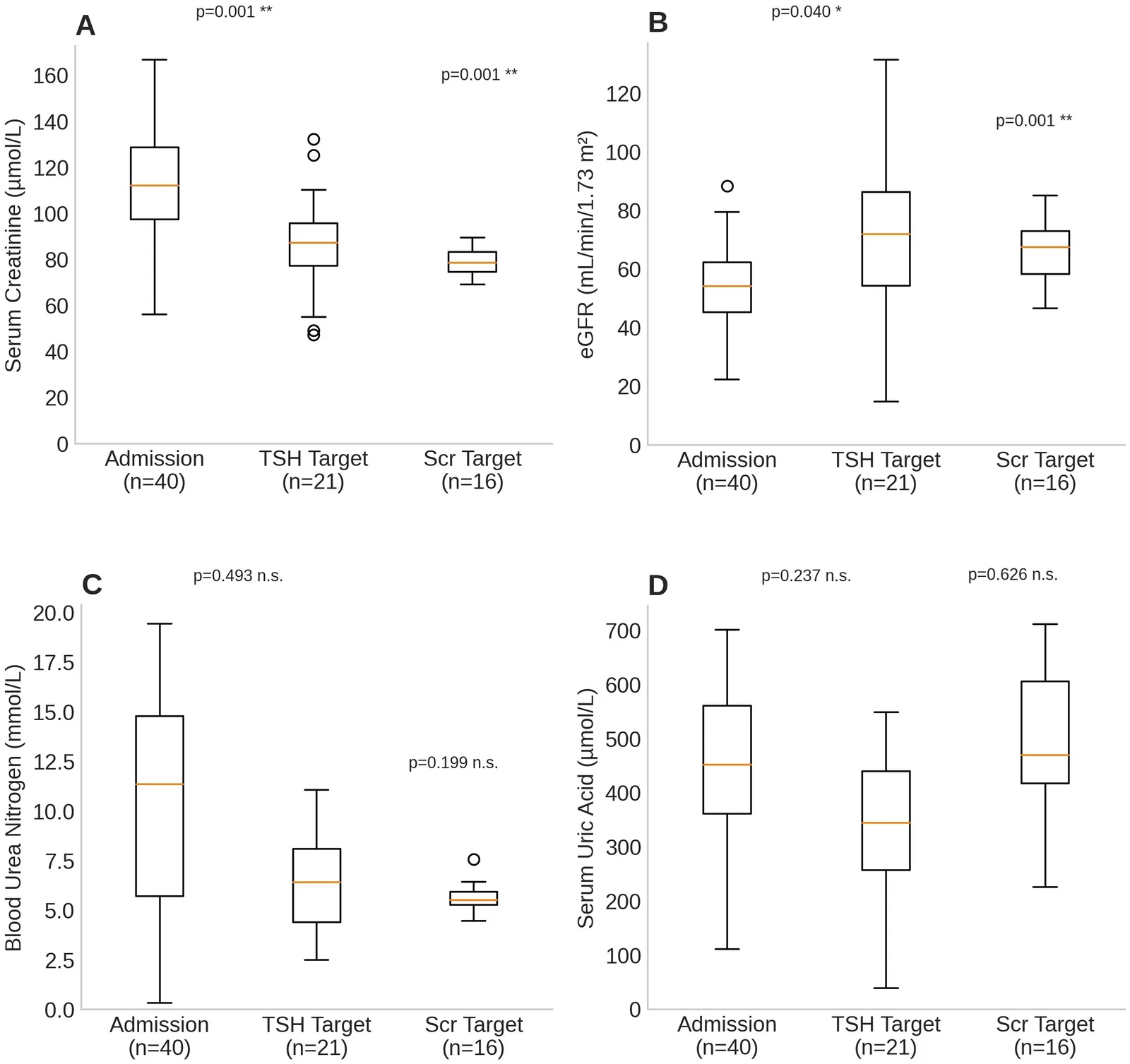
Renal function changes during treatment. **(A)** Serum creatinine, **(B)** estimated glomerular filtration rate (eGFR), **(C)** blood urea nitrogen (BUN), and **(D)** serum uric acid measured at admission (T0), TSH normalization (T1), and serum creatinine normalization (T2). Data are shown as boxplots. Serum creatinine was the primary renal outcome, whereas eGFR was evaluated as a supportive calculated indicator. P values were calculated using paired t-tests or Wilcoxon signed-rank tests, as appropriate according to data distribution. Analyses at T1 and T2 represent responder-based subgroups rather than the entire cohort.

At admission (T0), the mean serum creatinine (Scr) level was 114.88 ± 25.61 μmol/L, consistent with impaired renal function in this cohort of patients with newly diagnosed hypothyroidism. Following normalization of thyroid-stimulating hormone levels (T1), Scr declined significantly to 85.13 ± 21.01 μmol/L (P = 0.001), demonstrating a significant within-patient reduction compared with baseline. With further follow-up, Scr decreased to 79.92 ± 5.64 μmol/L at the time of Scr normalization (T2), representing a sustained and statistically significant reduction compared with baseline (P < 0.001). The mean absolute reduction in Scr from T0 to T2 was −34.96 μmol/L (95% CI: −52.1 to −17.8), indicating a clinically relevant change in creatinine levels (**Figure 2A**). Because analyses at T1 and T2 were restricted to patients who achieved predefined biochemical targets, these findings should be interpreted as observations within responder-based subgroups rather than longitudinal changes across the entire cohort.

#### Estimated glomerular filtration rate

At baseline, the calculated mean eGFR was 51.42 ± 16.32 mL/min/1.73 m², consistent with reduced estimated glomerular filtration. After TSH normalization, eGFR increased to 74.48 ± 32.56 mL/min/1.73 m² (P = 0.040), representing a significant change compared with admission. At T2, eGFR further improved to 75.04 ± 14.52 mL/min/1.73 m² (P = 0.001 vs baseline). The net increase in eGFR from admission to Scr normalization was +23.62 mL/min/1.73 m² (95% CI: +10.5 to +36.7), paralleling the observed reductions in serum creatinine (**Figure 2B**). Given that eGFR was calculated using the MDRD equation based on serum creatinine, these findings should be interpreted as supportive of the primary creatinine results rather than independent evidence of renal improvement.

#### Blood urea nitrogen and uric acid

Blood urea nitrogen levels decreased from 9.87 ± 5.14 mmol/L at T0 to 6.77 ± 2.08 mmol/L at T1 and 5.62 ± 1.12 mmol/L at T2; however, these changes did not reach statistical significance (P = 0.493 and 0.199, respectively) (**Figure 2C**). Serum uric acid levels showed a variable pattern, decreasing from 444.93 ± 175.38 μmol/L at baseline to 357.03 ± 137.67 μmol/L at T1, followed by an increase to 451.20 ± 141.93 μmol/L at T2 (**Figure 2D**). Thus, unlike Scr and eGFR, BUN and uric acid did not demonstrate consistent or statistically significant changes during short-term follow-up.

### Thyroid function indicators

#### Thyroid-stimulating hormone

**Figure 3:**
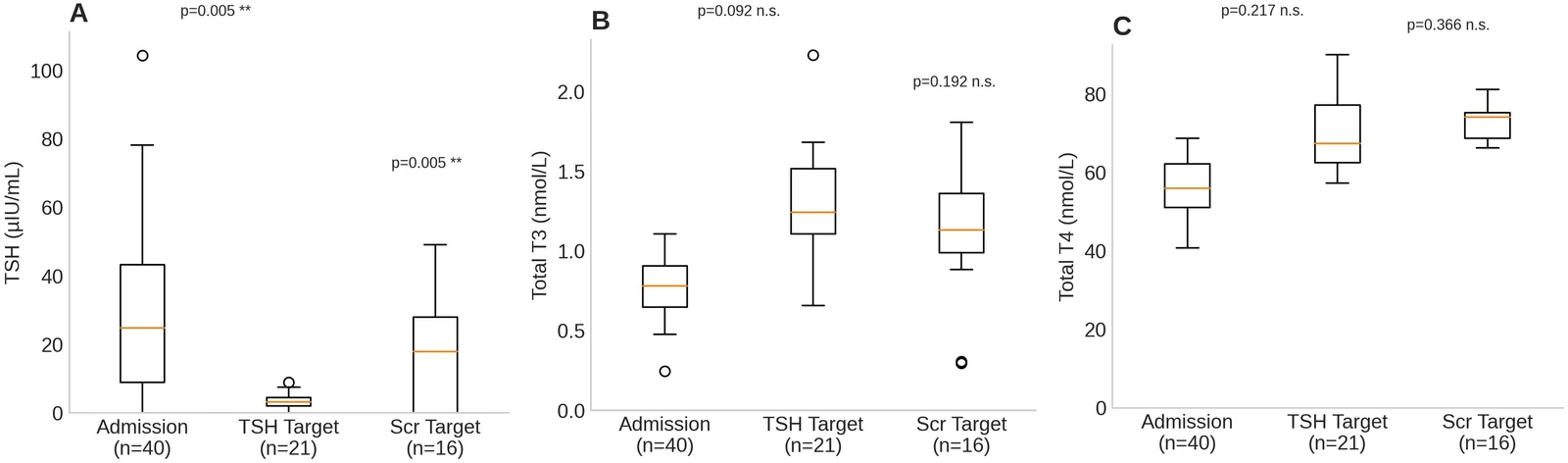
Thyroid hormone dynamics during treatment. Changes in **(A)** thyroid-stimulating hormone (TSH), **(B)** total triiodothyronine (T3), and **(C)** total thyroxine (T4) across admission (T0), TSH normalization (T1), and serum creatinine normalization (T2). Data are presented as boxplots. P values were calculated using paired t-tests or Wilcoxon signed-rank tests, as appropriate according to data distribution.

At admission, patients exhibited markedly elevated serum TSH levels. Following levothyroxine therapy, TSH decreased significantly at the time of target achievement (TSH target group), reaching near-normal values (P = 0.005). Although TSH levels showed a mild increase at the serum creatinine (Scr) normalization stage, they remained significantly lower than baseline values (P = 0.005) (**Figure 3A**). Total T3 levels demonstrated a modest upward trend after TSH normalization compared with admission; however, this change did not reach statistical significance (P = 0.092). At the Scr normalization stage, total T3 levels slightly declined relative to the TSH target stage, with no significant difference compared to baseline (P = 0.192) (**Figure 3B**). Total T4 levels increased progressively from admission through TSH normalization and Scr normalization; however, these differences were not statistically significant (P = 0.217 and P = 0.366, respectively) (**Figure 3C**).

#### Free thyroid hormones (FT3 and FT4)

**Figure 4:**
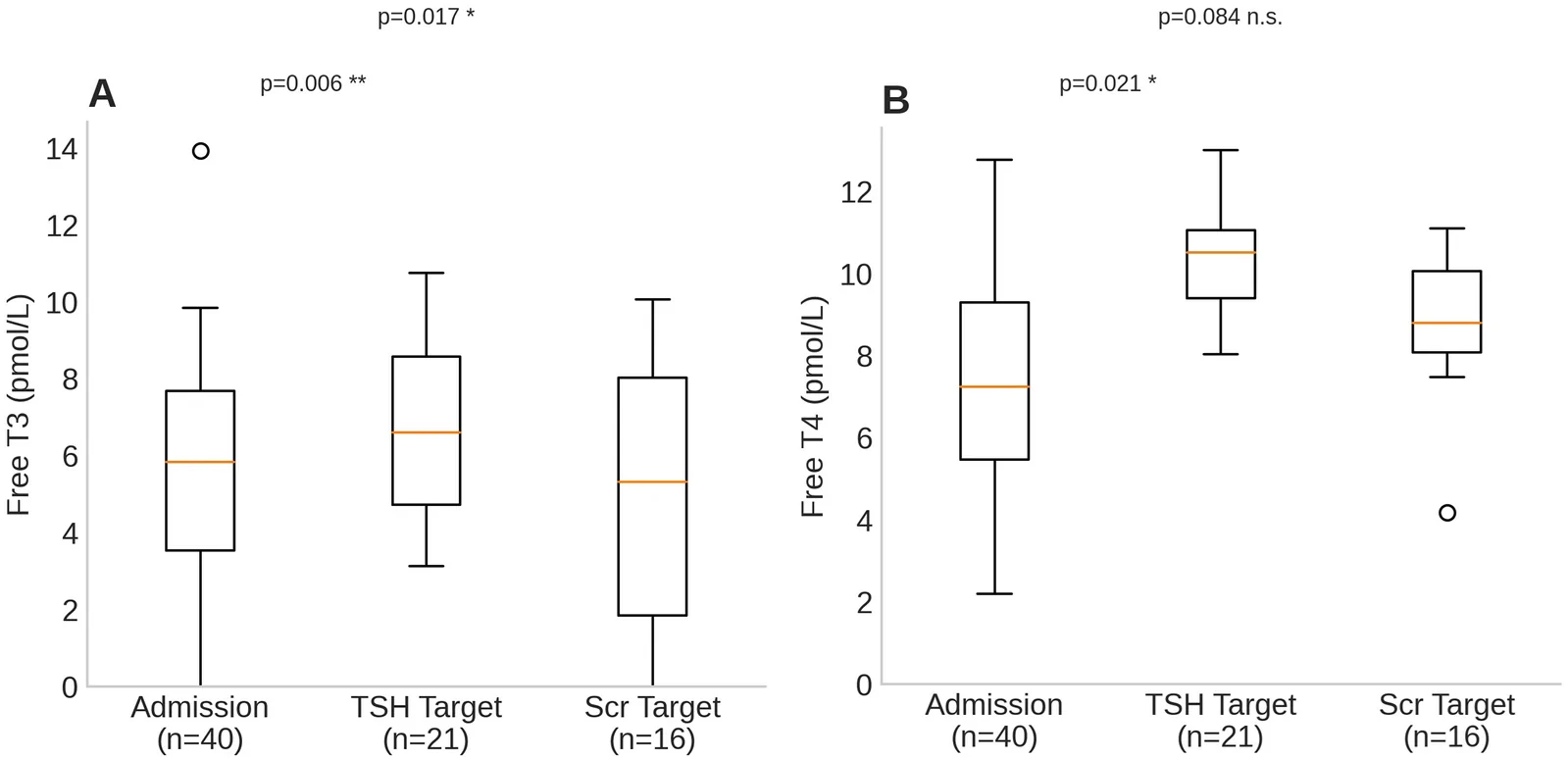
Free thyroid hormone changes. **(A)** Free triiodothyronine (FT3) and **(B)** free thyroxine (FT4) levels at admission (T0), TSH normalization (T1), and serum creatinine normalization (T2). Data are shown as boxplots. P values were calculated using paired t-tests or Wilcoxon signed-rank tests, as appropriate according to data distribution.

Free triiodothyronine (FT3) levels increased from 5.47 ± 3.37 pmol/L at baseline to 5.71 ± 2.65 pmol/L at T1 (P = 0.006), followed by a modest decline to 4.93 ± 3.10 pmol/L at T2, which remained significantly different from baseline (P = 0.017) (**Figure 4A**). Free thyroxine (FT4) increased from 7.10 ± 3.52 pmol/L at admission to 10.70 ± 1.18 pmol/L at T1 (P = 0.021) and was maintained at 9.22 ± 1.68 pmol/L at T2 (P = 0.084 vs baseline) (**Figure 4B**). Overall, free thyroid hormone levels demonstrated biochemical improvement during follow-up.

#### Liver and muscle enzymes

**Figure 5:**
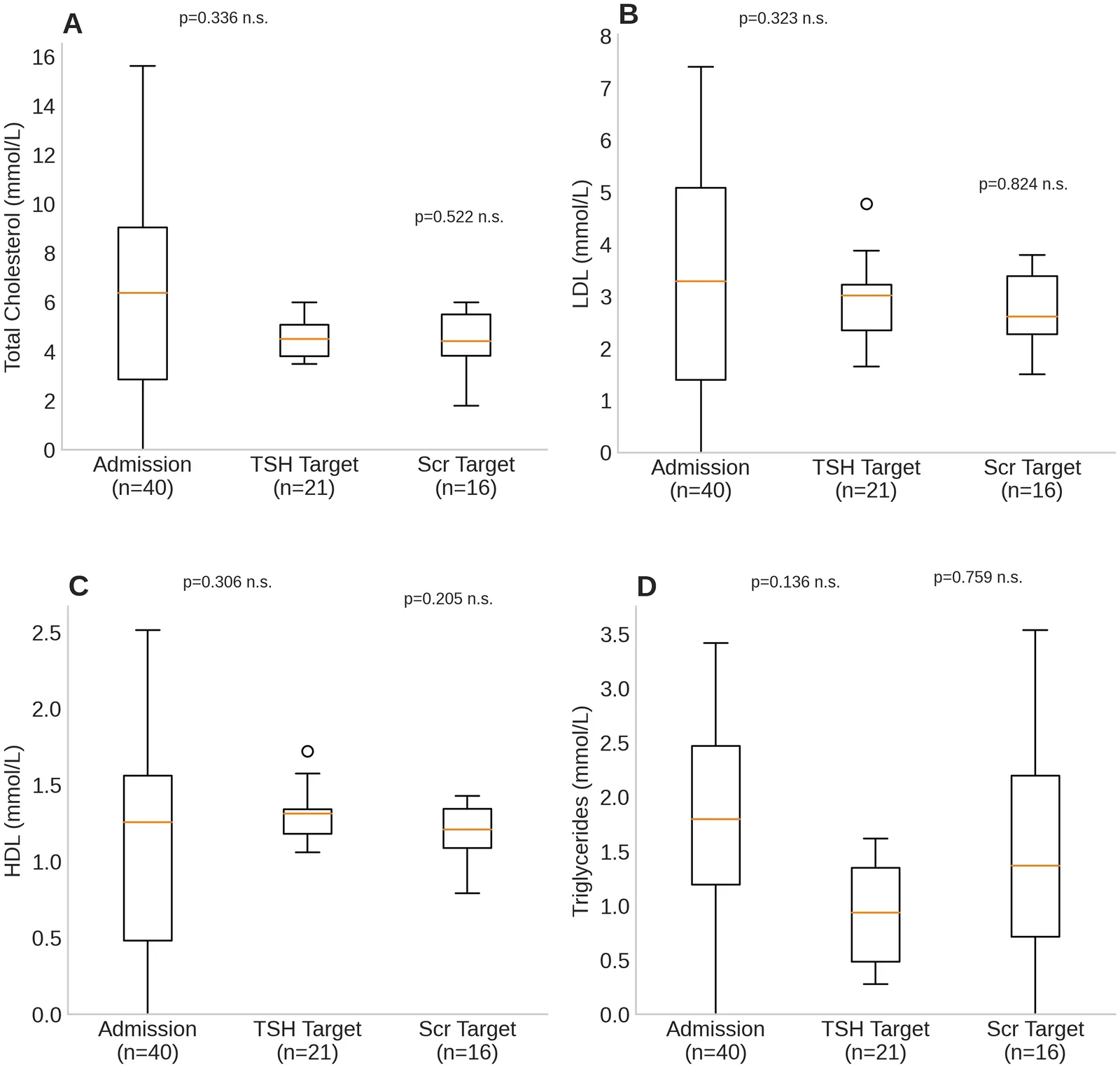
Hepatic and muscle enzyme profiles. **(A)** Alanine aminotransferase (ALT), **(B)** aspartate aminotransferase (AST), **(C)** creatine kinase (CK), and **(D)** creatine kinase-MB (CK-MB) levels across study timepoints. Boxplots display median, interquartile range, and outliers. P values were calculated using paired t-tests or Wilcoxon signed-rank tests, as appropriate according to data distribution.

No statistically significant changes were observed in alanine aminotransferase, aspartate aminotransferase, creatine kinase, or creatine kinase-MB levels between baseline and follow-up assessments (all P > 0.1). Enzyme values remained within or close to reference ranges throughout the study period (**Figures 5A–D**; **Table 2**).

**Table 2:** Cardiac function indicators at admission, TSH normalization, and serum creatinine normalization.

Indicator	Reference range	Admission (n=40)	TSH target (n=21)	Scr target (n=16)	P-value①	P-value②
Hemoglobin (g/dL)	13.5–17.5 (M), 12.0–15.5 (F)	12.8 ± 1.6	13.2 ± 1.4	13.4 ± 1.3	0.093	0.061
BNP (pg/mL)	<100	220 ± 85	150 ± 60	120 ± 55	0.048	0.022
Ejection Fraction (%)	55–70	48 ± 10	55 ± 8	58 ± 7	0.039	0.030
Pericardial effusion (cases)	0	3	1	0	–	–
Arterial sclerosis (cases)	0	5	3	2	–	–

Continuous variables are presented as mean ± SD and were compared using paired t-tests or Wilcoxon signed-rank tests according to data distribution. P-value①: Admission vs. TSH Target; P-value②: Admission vs. Scr Target.

#### Lipid profile

**Figure 6:**
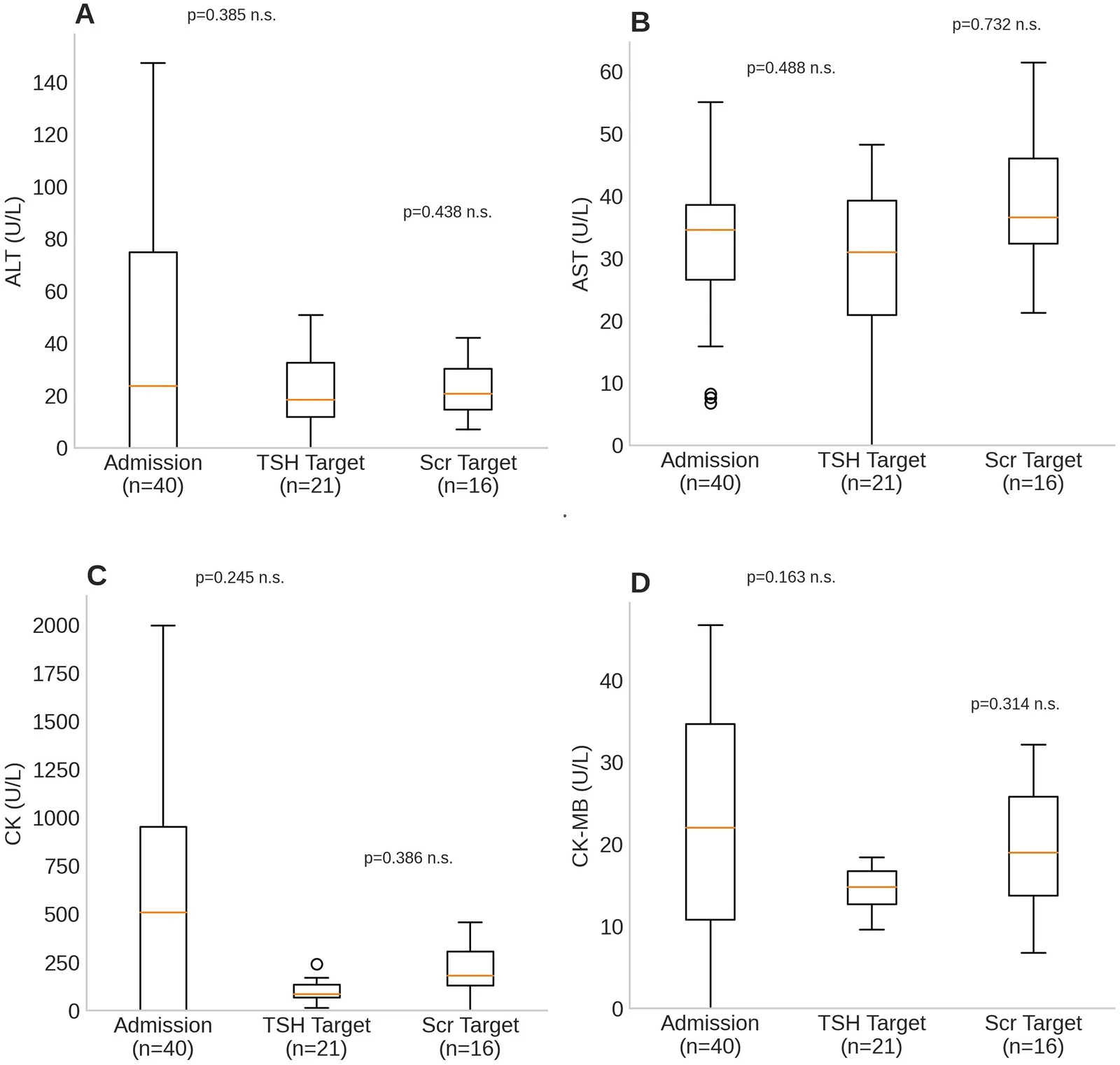
Lipid profile changes during follow-up. **(A)** Total cholesterol, **(B)** low-density lipoprotein cholesterol (LDL-C), **(C)** high-density lipoprotein cholesterol (HDL-C), and **(D)** triglycerides measured at admission (T0), TSH normalization (T1), and serum creatinine normalization (T2). Data are shown as boxplots. P values were calculated using paired t-tests or Wilcoxon signed-rank tests, as appropriate according to data distribution.

Serum triglycerides, total cholesterol, low-density lipoprotein cholesterol, and high-density lipoprotein cholesterol showed no significant changes across the three timepoints (all P > 0.1) (**Figures 6A–D**; **Table 1**).

#### Cardiac parameters

Hemoglobin levels showed a modest increase from 12.8 ± 1.6 g/dL at admission to 13.4 ± 1.3 g/dL at follow-up, although this change did not reach statistical significance (P = 0.093). In contrast, B-type natriuretic peptide levels decreased significantly from 220 ± 85 pg/mL to 120 ± 55 pg/mL (P = 0.022), and left ventricular ejection fraction improved from 48 ± 10% to 58 ± 7% (P = 0.030). Additionally, the frequency of pericardial effusion decreased from three cases at baseline to none at follow-up, and documentation of arterial sclerosis was less frequent at follow-up (**Table 2**). These observations describe changes occurring during hospitalization and should not be interpreted as being specifically attributable to levothyroxine therapy.

### Expanded patient characterization

Among the 40 patients, 31 (77.5%) were diagnosed with overt hypothyroidism and 9 (22.5%) had subclinical hypothyroidism. Autoimmune hypothyroidism (Hashimoto’s thyroiditis) was identified in 10 patients, while 4 patients had post-surgical hypothyroidism; the remaining cases were classified as other or unspecified causes of hypothyroidism. Autoimmune hypothyroidism was defined by the presence of positive thyroid peroxidase and/or thyroglobulin antibodies in conjunction with characteristic ultrasonographic findings. Thyroid peroxidase antibodies were positive in 28 patients and thyroglobulin antibodies in 26. Thyroid ultrasonography revealed hypoechogenicity in 24 patients and thyroid atrophy in eight. Common comorbidities included hypertension in 25 patients, diabetes mellitus in 15 patients, and mild chronic kidney disease (stage 1–2) in five patients. Proteinuria (1+ to 2+) was detected in four patients at baseline and resolved during follow-up. Glycemic control improved modestly, with HbA1c decreasing from 7.1 ± 1.2% to 6.4 ± 0.8% at T1 and 6.8 ± 0.9% at T2. Blood pressure also declined during follow-up (**Table 1**). These descriptive findings provide clinical context for the study population; however, because of the retrospective observational design, responder-based subgroup analyses, and potential influence of concurrent inpatient management, they should not be interpreted as evidence of causal effects of thyroid hormone replacement.

## Discussion

This study identified a clinically relevant association between hypothyroidism and improvement in creatinine-based renal indices during thyroid hormone replacement, underscoring the importance of early recognition and timely intervention. The significant reductions in serum creatinine and corresponding increases in estimated glomerular filtration rate observed during follow-up suggest that renal impairment associated with hypothyroidism may be predominantly functional rather than structural in this cohort. However, these findings should be interpreted within the context of a retrospective observational design and target-achievement subgroup analyses. These findings are consistent with previous mechanistic and clinical studies showing that thyroid hormone deficiency adversely affects renal hemodynamics and filtration, changes that have been reported to improve following restoration of euthyroidism (16–23). The magnitude of renal change observed in this cohort was clinically notable. Serum creatinine decreased from 114.88 to 79.92 μmol/L, while eGFR improved from 51.42 to 75.04 mL/min/1.73 m². These changes align with earlier reports demonstrating improvements in creatinine clearance and glomerular filtration following thyroid hormone replacement in diverse populations, including both Asian and Western cohorts (4, 16, 19). Furthermore, a recent systematic review and meta-analysis by Wang et al. (2026) reported that hypothyroidism is consistently associated with impaired renal function and that levothyroxine replacement is associated with improvements in serum creatinine and eGFR, although the certainty of evidence remains limited because most available studies are observational and heterogeneous (6). Collectively, these findings support the concept that hypothyroidism may represent a potentially reversible contributor to renal dysfunction, particularly in patients presenting with unexplained elevations in serum creatinine. However, these findings should be interpreted as associations rather than evidence of causality. From an etiological perspective, the predominance of autoimmune hypothyroidism in this cohort, supported by positive thyroid antibodies and characteristic ultrasound findings, is consistent with existing epidemiological data. Basu and Mohapatra previously highlighted autoimmune thyroiditis as a common link between thyroid dysfunction and renal impairment, mediated through immune, inflammatory, and hemodynamic pathways (18). Because no formal subgroup analyses comparing autoimmune and non-autoimmune hypothyroidism were performed, the present study cannot determine whether the observed renal changes differed according to disease etiology. In contrast to the consistent improvements observed in serum creatinine and eGFR, blood urea nitrogen and uric acid levels did not demonstrate uniform or statistically significant trends. These parameters are influenced by multiple nonrenal factors, including dietary protein intake, hydration status, and metabolic variability. Prior studies have also reported weak or inconsistent associations between uric acid levels and renal function in hypothyroid patients, supporting their limited utility as primary indicators of renal recovery in this setting (24). Accordingly, serum creatinine, with eGFR as a calculated supportive parameter, appears to be a more reliable marker for monitoring renal changes during thyroid hormone replacement.

Beyond renal outcomes, several systemic changes were observed. Hemoglobin levels increased modestly, consistent with partial correction of hypothyroidism-associated anemia following restoration of thyroid hormone levels (17). Improvements in blood pressure and selected cardiac function parameters, including reductions in B-type natriuretic peptide levels and increases in ejection fraction, were also observed during follow-up. However, because all patients received routine inpatient medical care, including optimization of blood pressure, glycemic control, fluid management, and other supportive treatments as clinically indicated, these improvements cannot be attributed specifically to levothyroxine therapy. The resolution of proteinuria in affected patients, although based on semi-quantitative dipstick assessment and limited follow-up duration, is consistent with reports suggesting that hypothyroidism may transiently alter glomerular permeability (3, 17). However, these findings may also be influenced by concurrent inpatient management and should not be interpreted as independent effects of thyroid hormone replacement. These findings should be interpreted cautiously given the absence of quantitative albumin measurements and long-term follow-up. In contrast, liver enzymes, muscle markers, and lipid profiles remained largely unchanged during the short observation period. This finding is consistent with previous studies indicating that lipid abnormalities and hepatic metabolic alterations associated with hypothyroidism may require prolonged therapy or adjunctive interventions to normalize (10, 11, 15, 25–31). The absence of short-term lipid improvement therefore likely reflects differing temporal dynamics of endocrine recovery across organ systems.

Several limitations of this study should be acknowledged. First, the retrospective observational design precludes strict control over follow-up intervals and laboratory testing schedules and limits causal inference. Second, the modest sample size and progressive reduction in the number of patients reaching predefined biochemical recovery stages (TSH normalization and serum creatinine normalization) reduced statistical power for later comparisons. Because follow-up analyses at T1 and T2 were restricted to patients who achieved specific biochemical targets, these timepoints represent responder subgroups rather than the entire cohort, introducing potential conditioning (responder) bias that may overestimate the magnitude of observed improvements. A repeated-measures framework including all participants at all timepoints was not applied, and therefore results at T1 and T2 should be interpreted as conditional upon target achievement. Third, although an exploratory multivariable analysis was performed in a subset of patients with complete data, the limited sample size and incomplete longitudinal data restrict the ability to fully adjust for confounding variables. Residual confounding related to comorbid cardiovascular and metabolic conditions cannot be excluded. Furthermore, unmeasured factors such as hydration status, inpatient fluid administration, medication adjustments, and treatment of concurrent medical conditions may also have contributed to changes in renal indices. Finally, the relatively short duration of follow-up limited assessment of long-term renal outcomes, durability of proteinuria resolution, and delayed metabolic effects such as lipid normalization. No formal adjustment for multiple comparisons was performed for secondary outcomes, and findings should therefore be interpreted as exploratory.

## Conclusion

In conclusion, in this single-center retrospective cohort, thyroid hormone replacement was associated with significant short-term improvement in serum creatinine and calculated eGFR in hospitalized patients with hypothyroidism. These findings suggest that renal impairment observed in hypothyroid states may be at least partially functional and potentially reversible in some patients. Improvements in creatinine-based renal indices may serve as early markers of renal response during endocrine correction. Accordingly, these findings should be regarded as hypothesis-generating and require confirmation in larger prospective controlled studies. Prospective studies with larger cohorts, longer follow-up, appropriate control groups, and adjustment for confounding variables are required to confirm these observations and to clarify long-term renal and metabolic outcomes.

## Data Availability

The original contributions presented in the study are included in the article/**Supplementary Material**. Further inquiries can be directed to the corresponding author.
