# Supplementary material for: Thyroid hormone replacement is associated with improvement in renal dysfunction in patients with hypothyroidism: a retrospective observational study
Source: Front Endocrinol (Lausanne). 2026 Aug 24;17:1840954. doi: 10.3389/fendo.2026.1840954 (PMC13547073; doi:10.3389/fendo.2026.1840954)
Supplement: Supplementary file 1 [file Table1.docx]

**Supplementary Table 1.** Exploratory multivariable linear regression analysis of factors associated with change in serum creatinine (ΔScr)

| Variable | β coefficient | 95% Confidence Interval | P value |
| --- | --- | --- | --- |
| Baseline serum creatinine | −0.48 | −0.75 to −0.21 | 0.002 |
| Age (years) | −0.12 | −0.35 to 0.11 | 0.298 |
| Sex (male vs female) | 0.09 | −8.42 to 8.60 | 0.812 |
| Hypertension (yes vs no) | 0.15 | −7.25 to 7.55 | 0.674 |
| Diabetes mellitus (yes vs no) | 0.18 | −6.80 to 7.16 | 0.603 |
